# Reliable reporting of Faradaic efficiencies for electrocatalysis research

**DOI:** 10.1038/s41467-023-36880-8

**Published:** 2023-03-01

**Authors:** Paul A. Kempler, Adam C. Nielander

**Affiliations:** 1 Department of Chemistry and Biochemistry and the Oregon Center for Electrochemistry, University of Oregon, Eugene, OR 97403 USA; 2 SUNCAT Center for Interface Science and Catalysis, SLAC National Accelerator Laboratory, 2575 Sand Hill Road, Menlo Park, CA 94025 USA

**Keywords:** Electrochemistry, Electrocatalysis

## Abstract

This Comment highlights the importance of Faradaic efficiency reporting and discusses methods for reliable measurements of Faradaic efficiency in the electrocatalysis field.

## Teaser

Electrocatalysis research is accelerated by measurements of reaction kinetics via electrical signals. When competing electrochemical reactions are present, the burden of proof is on the experimenter to connect these electrical signals to the assumed reaction of interest. Here, we highlight measurements of Faradaic efficiency to support claims of electrocatalyst activity, selectivity, and stability.

Electrocatalysis research is booming. Clean, inexpensive, and renewable electric power is increasingly available and is driving scientific interest in generating commodity chemicals and fuels from electricity. Improving our understanding of the fundamental principles of electrocatalyst design could unlock improved process efficiencies and reduce the burden of these reactions on electrical grids. Although electrochemical measurements allow total reaction rates to be monitored continuously via the electrical current flowing between two electrodes, this current offers incomplete information about the specific chemical reactions occurring at the electrode interface. For this reason, direct measurements of products are needed to keep the *chemistry* in *electrochemistry*.

Faradaic efficiency (FE) describes the overall selectivity of an electrochemical process and is defined as the amount (moles) of collected product relative to the amount that could be produced from the total charge passed, expressed as a fraction or a percent^1^. Even with careful preparation of the electrochemical cell, knowledge of the chemical reactions occurring at the interface is often assumed and incomplete—competing reactions, corrosion processes, or product crossover complicate the evaluation of current to various Faradaic processes and can lead to erroneous assignments of reactions. Thus, robust measurements of FE are imperative not only as a description of reaction selectivity but also to support claims of activity and stability.

Here we discuss the importance of reliable FE reporting and describe a set of standard methods for measuring the FE of an electrocatalyst. We focus on a representative subset of reactions, all requiring protons and relevant to electrified chemical manufacturing, for which FE near unity is critical for commercialization. These reactions include the hydrogen evolution reaction (HER), the oxygen evolution reaction (OER), the oxygen reduction reaction (ORR), the carbon-dioxide reduction reaction (CO_2_RR), and the nitrogen reduction reaction (N_2_RR). FE measurements are particularly important for reactions such as CO_2_RR and N_2_RR, where the competing HER cannot be ruled out on thermodynamic arguments alone. Luckily for laboratory scientists, many tools are available to quantify products, and modern mass spectrometry (MS) methods enable FEs to be measured in nearly real time.

## When should Faradaic efficiency measurements be expected for publication?

Thermodynamics provides a foundation for identifying potentially competing reactions in electrochemical cells. Standard reduction potentials, either measured or calculated from thermochemical data, allow a researcher to predict which reactions may occur at substantial rates at a given electrochemical potential. Table 1 presents the standard reduction potentials for a subset of reactions of interest to the electrocatalysis community. The measured current may be directly related to reaction rates without further analysis only when catalyst materials are thermodynamically stable and the cell is free of competing reactions and reactants (as defined by the potential of the working electrode). Notably, the ORR can interfere with nearly all other reduction reactions, requiring careful removal of O_2_ from both the reaction headspace and the electrolyte. Because proton sources are required for the reactions in Table 1, the HER may contribute substantially to the current whenever the potential is negative of the equilibrium hydrogen potential, as defined by local pH; FE measurements are essential in evaluating electrocatalysts for the CO_2_RR and N_2_RR.

**Table 1:** Standard potentials versus the normal hydrogen electrode (NHE), reactions, and commonly employed FE measurement techniques for the proton-coupled reactions discussed in this Comment^6,15,16^

Standard potential (*E*^o^)	Abbreviation	Reaction	Techniques for FE
1.23 V vs. NHE	ORR/OER	O_2_ + 4H^+^ + 4e^–^ **⇌** 2H_2_O	Gas collection, sensors^5^, GC
0.70 V vs. NHE	ORR	O_2_ + 2H^+^ + 2e^–^ **⇌** H_2_O_2(aq)_	UV-Vis, RRDE^9^, titration^3^
0.27 V vs. NHE	N_2_RR	N_2_ + 8H^+^ + 6e^–^ **⇌** 2NH_4_^+^_(aq)_	UV-Vis^2^, NMR, MS^7^
0.17 V vs. NHE	CO_2_RR (CH_4_)	CO_2_ + 8H^+^ + 8e^–^ **⇌** CH_4_ + 2H_2_O	GC, GC-MS^6^, DEMS
0.02 V vs. NHE	CO_2_RR (CH_3_OH)	CO_2_ + 6H^+^ + 6e^–^ **⇌** CH_3_OH + H_2_O	GC-MS^6^, DEMS, NMR^6^
0.00 V vs. NHE	HER/HOR	2H^+^ + 2e^–^ **⇌** H_2_	Gas collection^3^, sensors, GC
−0.10 V vs. NHE	CO_2_RR (CO)	CO_2_ + 2H^+^ + 2e^–^ **⇌** CO + H_2_O	RRDE^10^, GC, GC-MS, DEMS
−0.12 V vs. NHE	CO_2_RR (HCOOH)	CO_2_ + 2H^+^ + 2e^–^ **⇌** HCOOH_(aq)_	NMR^6^, LC-MS, RRDE

Metal corrosion poses long-term stability concerns and can lead to the spontaneous generation of H_2_ and other reduced products, which may lead to measured FEs greater than unity. The onset potentials for corrosion can also be predicted on thermodynamic grounds: for example, the corrosion potential of Co/Co^2+^ (*E*^o^ = –0.3 V vs. the normal hydrogen electrode (NHE)) implies that Co-based electrocatalysts are likely to be passive while facilitating the HER in O_2_-free 1.0 M OH^–^ (*E*^o^ = –0.8 V vs. NHE) but quantification of corrosion products is advisable if facilitating the HER in 1.0 M H^+^ (*E*^o^ = 0.0 V vs. NHE).

We provide the following guidance on when FE measurements should be required for publication, based on thermodynamic arguments:

- Could multiple products be formed at the applied potential at the working electrode?
- Are reactants/contaminants present in the cell environment (e.g., O_2_) that are competitive as defined by the applied potential at the working electrode (Table 1)?
- Are all components of the electrocatalyst and its support (carbon is of particular concern for OER) stable at the applied potentials and in the presence of other possible reactants and products (e.g., O_2_, H_2_O_2_, or H^+^)?

If multiple products, including possible corrosion products, are expected, additional chemical product characterization is a critical component of catalyst activity assessment.

## Batch methods for measuring Faradaic efficiency

**Fig. 1:**
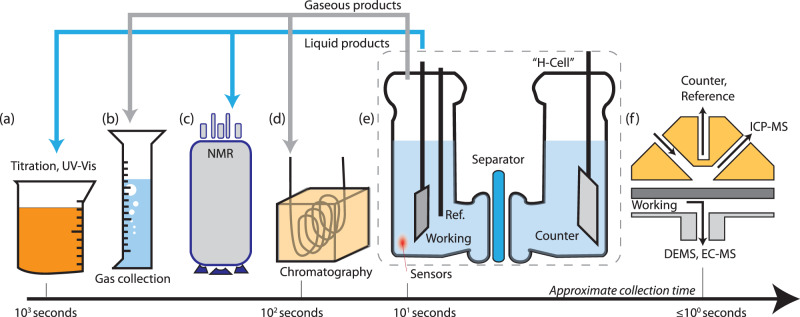
Instruments and methods for measuring Faradaic efficiency. Batch methods for characterizing gaseous and liquid products include titration (**a**), gas collection (**b**), NMR (**c**), and headspace characterization via gas chromatography (**d**). H-cells are used to separate the working and counter electrode for the collection of products (**e**). Electrochemical flow cells (**f**) enable nearly real-time detection of reaction and corrosion products via mass-spectrometry techniques (ICP-MS, DEMS, EC-MS).

Titrations (Fig. 1a) are a routine method for quantifying the liquid products of bulk electrolysis. Recently, colorimetric methods (e.g., indophenol, Nessler) have seen renewed popularity in measurements of NH_3_ production during the N_2_RR and other electrocatalytic NH_3_-producing processes^2^. In addition to direct analysis of the working electrolyte, researchers should first validate the sensitivity and extinction coefficient of the indicator in the presence of interferents. As a matter of practice, these colorimetric methods are not considered appropriate for monitoring trace products or when common laboratory contaminants can act as an interfering source^2^.

For gaseous products (HER, OER), selectivity has been monitored by measuring the volume of gas collected from the working electrode in an inverted burette or graduated cylinder (Fig. 1b)^3^. One potential pitfall in this method is that it implicitly assumes that products are entirely converted to the gas phase, whereas supersaturation of the electrolyte may lead to a lower-than-expected FE. Testing at current densities >10 mA cm^−2^ to drive gas nucleation at the electrode surface^4^, for a sufficient duration such that headspace products greatly exceed dissolved gases, and minimizing the volume of electrolyte that can collect supersaturated gases, will lead to more accurate FE measurements.

Properly calibrated electrochemical sensors (HER, OER, CO_2_RR) and fluorescence detectors (OER) in a well-stirred cell have been used to monitor the conversion of a reaction in near-real time, expressed as a rising product concentration in the electrolyte or headspace^5^. Gas chromatography (GC) and liquid chromatography (LC) are preferred for resolving dilute products produced at research-scale electrodes (Fig. 1d). When coupled with MS, GC-MS and LC-MS allow researchers to validate that products have been derived from isotopically enriched reactants (e.g., ^13^CO_2_ and ^15^N_2_)^6,7^. Isotopically enriched products have also been resolved using NMR (Fig. 1c)^7^ to discriminate between contaminant and electrocatalytically produced N_2_RR products.

Researchers must validate their ability to accurately sample the phases containing electrochemical products; errors introduced via sampling are nearly always greater than the standard error of the instrument. The inclusion of error bars, representing the standard deviation of at least three separate FE measurements, provides a more direct assessment of the measurement reproducibility. Loss of products via crossover between the working and counter electrode will lead to lower measured Faradaic efficiencies. This effect can be mitigated, but not prevented, by separating electrodes with a semipermeable membrane. For flow-through measurements, improper estimation of gas flow is commonly observed for the dissolution of CO_2_ into aqueous, alkaline electrolytes, which leads to greater-than-expected concentrations of products and overestimations of the FE^8^. Precise downstream measurements of volume flow, near the instrument sampling product concentrations, are recommended. When the sum of all product FEs is significantly less than 100%, researchers may investigate escaped products, consumption at the counter electrode, and homogeneous reactions within the cell; total FEs greater than 100% may be due to overestimation of sampled volume, sampling a preconcentrated product, or spontaneous generation of product through a chemical reaction (e.g., corrosion).

## Real-time methods for measuring Faradaic efficiency

Direct collection of electrolyte volumes near the electrode surface can enable a continuous analysis of products such that the measured FE approximates the current efficiency (CE). We endorse a textbook distinction of CE, which refers to the instantaneous selectivity toward a specific half-reaction^1^, although FE and CE have often been used interchangeably. For kinetically facile 2e^–^ reactions, rotating-ring disc electrodes (RRDE) combine a generator electrode (the disc) with a collector electrode (the ring) to simultaneously measure reaction rates and product selectivity at equivalent time scales. This method has been classically used for quantifying the FE between the 2e^–^ and 4e^–^ pathways of the ORR^9^ (Table 1) and, more recently, was used to monitor the FE and local pH shift during CO_2_RR at an Au surface^10^. The collection efficiency of the ring should be carefully calibrated using a one-electron redox couple (e.g., Fe(CN)_6_^4^^–^/Fe(CN)_6_^3^^–^) in the supporting electrolyte of interest.

MS tools coupled to electrochemical flow cells (e.g., differential electrochemical MS [DEMS] or electrochemical MS [EC-MS], Fig. 1f) improve temporal resolution in FE measurements by detecting changes in the mass-selected signal of products collected adjacent to the working electrode surface. Because the relationship between ion current and FE is itself ambiguous, careful reporting of the calibration method (either via standard samples or a cell environment with FE ~ 1) is important. This method has been used to simultaneously measure hydrogen, hydrocarbons, and oxygenates produced at a Cu thin film^11,12^ and has also been used to monitor corrosion reactions at carbon supports^13^. For aqueous corrosion products, online inductively coupled plasma MS (ICP-MS, Fig. 1f) has correlated corrosion data within seconds of changes in the applied potential^14^. Such measurements are critical for assessing the commercial viability of catalysts free of (or with low loadings of) platinum group metals and researchers measuring Faradaic efficiencies should not restrict their focus toward measurements of product selectivity but rather all of the electrochemical reactions occurring at the interface of interest.

## Data reporting

Transparency in electrocatalysis research is critical and is supported by detailed descriptions of the methods used to measure FE. Reporting reactor volumes and sampled volumes for batch measurements and flow rates leaving a reactor for online measurements are good practices to allow readers and reviewers to independently assess the measurement. Control experiments using electrocatalysts with established activity and selectivity and/or labeled reactants are effective for measuring reproducibility and sources of systematic error.

In summary, the burden of proof is on the researcher to connect ambiguous electrochemical data to the assumed chemical reaction of interest. Inexpensive approaches involving titrations and gas collection are sufficiently accessible that some form of FE measurement should be connected to any electrochemical current that may be assigned to more than one half-reaction. Through hydrodynamic control of the electrolyte at the electrocatalyst surface, modern FE measurements now approach real-time quantification of products, facilitating more-robust reporting of catalyst activity, selectivity and stability.
